# Indian Psychiatry and *Indian Journal of Psychiatry* - A journey

**DOI:** 10.4103/0019-5545.69194

**Published:** 2010-01

**Authors:** T. S. Sathyanarayana Rao, G. Swaminath, G. Prasad Rao

**Affiliations:** Professor and Head, Department of Psychiatry, JSS University, JSS Medical College, Mysore - 570 004, India; 1 Professor of Psychiatry, Dr. B R Ambedkar Medical College, Kadugondanahalli, Bangalore - 560 045, Karnataka, India; 2 Director, Schizophrenia and Psychopharmacology Division, Asha Hospital., Banjara Hills. Hyderabad, India

*“When we climb high places of the Earth, plodding slowly at a mountaineer’s pace with crampons on our boots, that we may keep foothold on the blue ice, we should stop from time to time and, steadying ourselves with our ice-axe for a moment, raise our down-bent eyes, weary with guiding our steps between crevasses, to the great peaks, we would conquer, and see, too the foothills we have left behind. Only by gazing, thus, can the Alpine climber find values and often he catches too a glimpse of beauty.”*[1]

The Indian Journal of Psychiatry (IJP) in its multiple avatars has completed 60 years of existence. For an Institution like the Indian Psychiatric Society with IJP at its core, this is relatively a very small step. It has many miles to go and many more milestones to surpass.[2] However, it is time for us, like the alpine climber to “raise our eyes to the great peaks and also to the foothills we have left behind”. The IJP is a product of the creative labor of many distinguished past editors who have diligently toiled to bring the IJP to where it is today.[2] The journal has striven to reflect the evolution of psychiatry in India.

## THE BEGINNING-INDIAN JOURNAL OF NEUROLOGY AND PSYCHIATRY (1949-1954)

The dawn of Indian independence also saw the birth of the Indian Psychiatric Society (IPS) and may be regarded as the beginning of the modern psychiatric movement in the country.[3] In the second Annual General Body meeting of the IPS at Allahabad on January 1st and 2nd 1949, it was decided to bring out an official publication of the IPS under the editorship of Nagendra Nath De in the name of “Indian Journal of Neurology and Psychiatry,” ushering a new era.[4] The journal was a huge success in its inaugural year with four issues published well on time from Kolkata.

From the very beginning the IJP followed patterns used in reputed international journals containing sections such as editorial, original articles, reviews news and abstracts. De (1949-1951) in his first editorial dealt with the opportunities in psychiatric research in India, opening his remarks with, “Research is the life of Science. A Science is living only as long as research is carried out in it.”[5]

The first year had an extensive review article titled “Mental Health Services in India” by N. N. De. Most of the papers published then dealt with theoretical issues with a strong inclination towards psychoanalysis and other disciplines of psychology. The referencing style conformed to that used in Quarterly Cumulative Index Medicus.

De resigned on grounds of ill health and L. P. Varma. (1951-1954) took over as Editor. Varma edited three volumes of the journal, bringing out a total of nine issues, some of which were combined (Vol. 4: Issues 1 and 2, 3 and 4). Among the outstanding works done during this period were excellent reports on the phenomenology of general paresis of insane;[6] interesting observations on the relationship of leprosy with mental disorders;[7] two reports of the results of transorbital leucotomy[8,9] appearing within five years of the introduction of this technique by Freeman in 1949; a therapeutic trial of ‘mysoline,’ with patients serving as their own controls done within two years of the introduction of the drug[10] and electro-encephalographic study of the role of barbiturate anesthesia in patients receiving ECT.[11]

After Varma, M. V. Govindaswamy was elected its editor (1954-1958), but the journal ceased publication[4] due to dearth of good research papers mainly attributed to the backwardness of medical research in the country, lack of manpower (only around 80 psychiatrists by 1950) and lack of funds.[4] The last issue of Indian Journal of Neurology and Psychiatry (Vol. 5, Issue 1) appeared in 1954.

## THE INDIAN JOURNAL OF PSYCHIATRY-TOWARDS SILVER JUBILEE (1958-1984)

The revival of the Indian Journal of Psychiatry in its present name was decided at the annual meeting of the IPS in 1958 at Poona under the leadership of I.K. Mujawar. A council under M. V. Govindaswamy was to undertake necessary actions to its resumption. However Dr. M. V. Govindaswamy resigned as elected editor and Col P.N. Bardhan (1958-1960), a pathologist, became the editor and the first issue of the “Indian Journal of Psychiatry” was printed in October 1958 (Vol. 1, No. 1).

In the first editorial of the IJP, Col. Bardhan wrote, “The first series was called Indian Journal of Neurology and Psychiatry and there is significance in the change of the name of the journal. Both neurology and psychiatry are major specialties in their own right and a separate journal could easily be devoted to each. The first series was discontinued owing to unavoidable reasons, ill health none the least” and concluded with “the field of psychiatry in this country is very vast and ever expanding. Therefore, to justify its existence, this journal must represent the experience and progress in every branch of psychiatry”.[4]

The journal followed the same format as the earlier journals, but the referencing pattern changed to Harvard type and a supplement continued to accompany the first issue of the journal which included details about the meetings and members of various committees of the society.[4]

The first 25 years of the IJP mirrored the growth of psychiatry and there were many factors which influenced improved research and publication environment in the country.[3] A remarkable increase in the number of trained personnel available seems to have played a key role: the number of qualified psychiatrists in the country was nearly 100 in 1960 and it swelled up to nearly 400 in 1972.[3] Among other related factors were the emergence of a greater number of psychiatric units at the general hospital level and in the undergraduate medical college, much increased financial support for psychiatric institutions and the recognition of the importance of psychiatry by bodies like Indian Council of Medical Research.[3] The country’s general progress in the scientific sphere and greater awareness of the potentials of research, as a whole have surely contributed to this enthusiasm among the workers in the field of mental health.[3]

The IJP has since been in the forefront of publishing research by Indians and helped stimulating psychiatric thinking and research in the country.[4] It has, like most Psychiatric journals, been “both the repository and means of dissemination of professional learning as well as permanent records of facts which represent the growing horizon of psychiatric wisdom and progress”.[5]

Outstanding work was done during this phase in the field of epidemiology, phenomenology, and therapeutics. The focus of interest seems to have shifted from individual to mass problems and from man to society in general. There were excellent surveys of mental morbidity in general population by researchers from all over the country.[12–16] Large samples of patient population attending various psychiatric clinics were analyzed on clinical and demographic variables to establish correlations between social class and mental illness.[17–20] On a somewhat different plane this increased social consciousness is manifested by workers who undertook study of such varied social phenomena as student failure[21] as well as industrialization and migration.[22,23] The enormous amount of useful research data put forward on the psychological aspects of family planning and contraception[24–28] is yet another evidence of the intense social consciousness of the Indian psychiatrists in those days.

This remarkable interest in sociology of psychiatric disorders and in psychology of social phenomena may be attributed to the general enthusiasm, among the psychiatrists for action. This was coupled with either of the following two factors, namely: lack of resources to conduct laboratory research of any kind as well as the effect of changing foci of interest and attention from couch to the community in Western psychiatric world.[3]

The mistaken impression that epidemiological work is easy to undertake and quick to bear results may also have played some role in shifting the interest towards mass studies. The changed political atmosphere of the country and socialistic policies of Indian Government may also be responsible, though on a somewhat unconscious level, for this shift of attention.[3] In addition, very promising phenomenological studies were done on depression[29] and on schizophrenia.[30] On the therapeutic front, drug trials started gaining momentum. Most of these trails were of double blind variety and employed Controls; examples being on the use of Flupenthixol,[31,32] Fluphenazine enanthate,[33] Lorazepam,[34] and Trimipramine.[35] Newer techniques of other physical methods of treatment and Cingulotomy[36] have also been tried, though the patient samples here were not large enough for valid conclusions. This is of course understandable for the last mentioned treatment modality. Establishment of norms of performance of Indian population on certain psychological tests[37,38] and designing some original psychometric tools[39] were among other outstanding works done in this phase. The importance of having our own psychological tests, which have been designed specially for the purpose of Indian patients, cannot be over-emphasized.[3]

A few interesting papers on the relationship of crime with mental disorder[40–43] have also appeared during this phase. Psychiatrists serving in the armed forces of the nation have also contributed to this pool of psychiatric research endeavors; a notable study being- “Morale of battle casualties”.[44] The quality of research work was multidimensional though some topics such as Child Psychiatry and mental retardation remained virtually unexplored.[3]

Something that becomes quite obvious while reviewing the outstanding works, especially the ones in the second phase, is the preoccupation and attempts at, what one may call, “Indianization” of Psychiatry, Phenomenological studies with a tendency to explain the form and content of symptomatology in relation to Indian culture,[45] for example, Hysterical Psychosis,[46] Possession States,[47] Keemam dependence,[48] and Cannabis psychosis.[49] There were attempts at evolving an Indian classification of psychiatric disorders;[50–52] introduction of psychometric tools designed by our own workers;[39] utilization of certain mythological concepts in psychotherapeutic process;[53] and yoga in treatment of many neurotic and psychosomatic problems[54] were all pointers towards this urge for Indianization of Psychiatry. More and more workers, it appears, have started feeling that practice, teaching and research of psychiatry in India should be different from that in West.

These epidemiological, phenomenological and therapeutic studies, which attracted researchers in the 60s and 70s continued well into the 80s.[55] In addition, clinical studies formed a bulk of research in this period, most of the work being on depression and schizophrenia.[55]

This initial phase saw a total of four editors viz. Col P. N. Bardhan, (1958 -1960), M. R. Vachha, (1961-1967), Venkoba Rao (1968-1976) and B. B. Sethi (1977-1984), who was, supported by Rudraprakash as assistant editor. Col Bardhan was a pathologist, Vachha a neurologist and Venkoba Rao the first psychiatrist to be editor of the IJP. The IJP crossed significant milestones during their tenures.

In 1961, the IPS got a new emblem with a brain and a staff of Aesculapius carved on a potter wheel with *Prashanti* written in Hindi characteristics.[4] Under the leadership of Venkoba Rao the journal got a new look and new sections were added as per subjects of review articles. In 1973, the journal got indexed in Excerpta Medica which opened new vistas for international circulation of the journal.[4] Under the leadership of Sethi, in 1983, the journal was provided an International Student’s serial number (ISSN No. 0019-5545).[4] The same year the journal celebrated its silver jubilee with the gradual increase in the size of journal which swelled from 69 pages in 1958 to 400 pages in 1983.[4]

## TOWARDS THE GOLDEN JUBILEE (1985-2006) AND BEYOND

In this period there were a total of eight editors with three of them lasting for less than a term of two years. This churning was more in the last five years of this period. The teams of earlier editors and assistant editors were S. M. Channabasavanna (1985-1988) with S. Chaturvedi, A K Agarwal[56] (1989-1992) with J. K. Trivedi, K Kuruvilla (1993-1997) with M. Rajagopalan followed by J K Trivedi (1998-2002), all of whom had multiple terms. In 2003, U. Goswami became editor along with U. Khastagir and U. Kumar as assistant editors, but could not complete his term for personal reasons; T. S. S. Rao became the interim editor with B N Raveesh in 2004. N Desai (2005-2006) along with S. Mehrotra completed their two year term. T. S. S. Rao with G. Swaminath became editor in 2007 but as the IPS elections were annulled G. Swaminath became the interim editor for the latter part of 2007. Since 2008 T. S. S. Rao with G. Swaminath have been at the helm of IJP.

There have been many changes in the IJP over these 25 years. The journal has shown a steady growth and increase in readership. In the early nineties, author indexing was started and in 1994 subject index was added. In 1992 there was a distinct change in format of the text as type setting was changed. In 1993 (Vol 35. No. 1), the front page was again changed and the current format was used for the first time. Under J. K. Trivedi, the IJP was available in electronic format and linked to the newly formed website of the IPS. Under N. G. Desai, the IJP underwent major changes in the format which is still being followed. The referencing style of the journal was reviewed again and for the first time the Vancouver System was adopted.

Schizophrenia, major depression, substance abuse, organic disorders, psychiatric aspects of medical disorders and anxiety disorders respectively formed a major part of the publication in the IJP during this period.[57,58] The articles included biology and clinical studies equally in the initial part of this phase,[57] and in the last 10 years of this phase biological studies predominated.

Studies on schizophrenia concentrated on phenomenology,[58–60] course and outcome of schizophrenia,[61] deficit schizophrenia,[62] disabilities[63,64] rehabilitation[65] and quality of life. In keeping with the global trend there were many studies on biochemical, immunological, radiological and genetic issue[66,67] related to schizophrenia, and this was reviewed in the IJP in 2004.[68]

Studies on Depression and Bipolar Disorder too have concentrated on phenomenology,[69] course,[70–72] disability and quality of life.[73] In addition to articles on anxiety disorders,[74,75] social anxiety disorder[76] and obsessive compulsive disorder[77] have engaged the interest of researchers.

In comparison to the earlier quarter century, the number of articles on the elderly[78] has increased, which is in keeping with increased life expectancy. The IJP brought out a special supplement on dementia, which contained three editorials and 15 articles by researchers from all over the world. This supplement on dementia (IJP Vol. 51, Issue 5, January 2009) was released during ANCIPS 2009.

Studies on Addiction medicine, especially alcohol[79–81] as well as sexual medicine, more so in the Indian setting,[81–84] increased towards the latter part of this phase. There were demands for a separate subspeciality in the IPS for these areas.[85–87] The focus of Indian researchers has rightly moved on to psychological dysfunction,[88] life events,[89] disability,[90] family burden and participation,[88,91] rehabilitation,[65,92] as well as the impact on quality of life[93] in various psychiatric illnesses.

While drug trials predominated in the first 25 years, these dwindled during the next phase. Most drug trials were replications of those from the West, but helped evaluate efficacy and adverse effects in the Indian population.[94–99] However, the focus was on prescription practices,[100,101] drug adherence,[102] and pharmacoeconomics.[103,104] Other treatment modalities gained renewed interest. There were many articles on ECT which focused on the machine, methodology and practice.[105–107] Psychotherapy and its training too gained importance over the past two decades.[108–110] There were a few studies on epidemiology,[111,112] crime and mental disorder.[113]

Psychiatric education[114] has attracted the attention of academicians and in keeping with this need there was a special symposium on undergraduate psychiatric education eight articles with perspectives from UK, USA and India; articles were published in IJP (2007) Vol. 49 Issue 3.

## THE ELECTRONIC ERA (2007 ONWARDS)

The year 2007 saw three major transformations, all interrelated, in the journal. M/s Medknow Publications was appointed publisher, printer and distributor of the IJP who gave the journal access to the other two changes. An online manuscript submission and electronic peer review system (www.journalonweb.com) is now in place. This has reduced clerical work as well as submission-decision time and thanks to this system we now have about more than 200 articles in various stages of review or publication.[2]

One of the important advantages of the system is that the search for reviewers who are key researchers in the field can be identified from the references quoted in the article as well as a PubMed search using key words. Getting articles reviewed by such significant authorities has been an enriching experience which has greatly improved the worthiness of the publication.[2] It is important to bear in mind that the review process by these key researchers leads to speedier and better quality of review, with more people being aware of the journal. Some worthy referees volunteer to become authors and further enhance the richness of our journal.[2]

The second important transformation is the posting of the journal well before printing on the website (www.indianjpsychiatry.org) which contains full text articles has helped avoid the time lag between decision and printing and the articles have been available for viewing well before it arrives in the hands of subscribers.[2]

Some believe that there should not be open access to our articles, but the advantage of open access bestows on us greater visibility, and increased citations resulting in higher impact rates. The IJP celebrated its golden jubilee by holding an exhibition at Annual National Conference of the IPS (ANCIPS 2008) at Kolkata, which was well appreciated. The release of the IPS Golden Jubilee Commemorative volume which contained 50 presidential addresses and 50 editorials was another important feature. This volume was greatly cherished by members[115] as it gave a glimpse of the opinions, wisdom as well as vision of those at the helm of the IPS over the 50 years. The volume, however, missed out on some presidential addresses which could not be procured.

In 2009, a mammoth task of digitalizing all past issues of the Indian Journal of Neurology and Psychiatry (1949-1954), the Indian Journal of Psychiatry (1958-2008) was successfully undertaken and a DVD was distributed to all members during ANCIPS in January 2009 as well as archived in the IJP website www.indianjpsychiatry.org. The DVD, in addition, contained the Clinical practice guidelines of the IPS, as well as the updated membership directory of the IPS. The IJP now has its articles available “full text online, fully archived”.[116]

Thanks to these transformations, the journal is now indexed with various indexing agencies such as SCOPUS, DOAJ, Index Copernicus, Health and Wellness Research Center, Health Reference Center Academic, InfoTrac One File, Expanded Academic ASAP, Genamics JournalSeek, Ulrich’s International Periodical Directory, EBSCO Publishing’s Electronic Databases and Google Scholar.[117] The recent feather in the cap has been the acceptance of IJP in Pubmed.

The easy and universal access of all articles of the IJP from 1949 till date is a mine of information and this volume - “Annotations of Indian Psychiatry” is a natural extension. This volume is a compilation of articles on important research and publications by Indians, provides an insight to the achievements as well as the future of Indian Psychiatry.

The IJP still has a long way to go as we await the impact factor rating from Thomson Science Citation Index. We do hope this will come sooner than later and with a good weightage.

## CONCLUSION

Psychiatric journals are both the means and repository of dissemination of professional learning.[118] Journals are a permanent record of living facts-they represent the growing edge of psychiatric wisdom and progress.[118] The Indian Journal of Psychiatry has been a true reflection of the progress made by Indian psychiatrists in the field of psychiatric research.[57] IJP publications of editorials, presidential addresses and orations reflect the contemporary thinking of Indian psychiatrists as well as the Indian Psychiatric Society as a whole.[57]
